# Involvement of Vacuolar Processing Enzyme CgVPE1 in Vacuole Rupture in the Programmed Cell Death during the Development of the Secretory Cavity in *Citrus grandis* ‘Tomentosa’ Fruits

**DOI:** 10.3390/ijms241411681

**Published:** 2023-07-20

**Authors:** Bin Huai, Minjian Liang, Junjun Lin, Panpan Tong, Mei Bai, Hanjun He, Xiangxiu Liang, Jiezhong Chen, Hong Wu

**Affiliations:** 1 State Key Laboratory for Conservation and Utilization of Subtropical Agro-Bioresources, Guangdong Laboratory for Lingnan Modern Agriculture, South China Agricultural University, Guangzhou 510642, China; 18819492852@163.com (B.H.); yfdfh96@163.com (M.L.); yxxxljj2022@163.com (J.L.); tongpanpan0227@126.com (P.T.); baimei924@scau.edu.cn (M.B.); hhjmnxy@scau.edu.cn (H.H.); liangxiangxiu@scau.edu.cn (X.L.); 2 Guangdong Technology Research Center for Traditional Chinese Veterinary Medicine and Natural Medicine, South China Agricultural University, Guangzhou 510642, China; 3 College of Horticulture, South China Agricultural University, Guangzhou 510642, China; cjzxb@scau.edu.cn

**Keywords:** CgVPE1, vacuole destruction, programmed cell death, secretory cavity

## Abstract

Vacuolar processing enzymes (VPEs) with caspase-1-like activity are closely associated with vacuole rupture. The destruction of vacuoles is one of the characteristics of programmed cell death (PCD) in plants. However, whether VPE is involved in the vacuole destruction of cells during secretory cavity formation in *Citrus* plants remains unclear. This research identified a *CgVPE1* gene that encoded the VPE and utilized cytology and molecular biology techniques to explore its temporal and spatial expression characteristics during the PCD process of secretory cavity cells in the *Citrus grandis* ‘Tomentosa’ fruit. The results showed that CgVPE1 is an enzyme with VPE and caspase-1-like activity that can self-cleave into a mature enzyme in an acidic environment. *CgVPE1* is specifically expressed in the epithelial cells of secretory cavities. In addition, it mainly accumulates in vacuoles before it is ruptured in the secretory cavity cells. The spatial and temporal immunolocalization of CgVPE1 showed a strong relationship with the change in vacuole structure during PCD in secretory cavity cells. In addition, the change in the two types of VPE proteins from proenzymes to mature enzymes was closely related to the change in CgVPE1 localization. Our results indicate that CgVPE1 plays a vital role in PCD, causing vacuole rupture in cells during the development of the secretory cavity in *C. grandis* ‘Tomentosa’ fruits.

## 1. Introduction

Programmed cell death (PCD) is widely involved in plant development processes, such as vegetative and reproductive processes [1]. Some morphological and biochemical changes have been observed to be similar in plant PCD and animal apoptosis, in addition to some conserved regulatory mechanisms [2,3]. However, plants have specific vacuoles and cell walls that complicate the PCD process. Recently, vacuoles have been increasingly recognized for their role in cellular signaling during the regulation of cell death [4]. Studies have indicated that vacuoles play an important role in PCD, including hypersensitivity reactions (HR) caused by pathogens [5], developmental cell death [6], and the formation of plant tracheary elements (TEs) [7]. Plant vacuoles can contribute to PCD in destructive and nondestructive ways, and developmental PCD usually occurs in a destructive way. Destruction is caused by tonoplast rupture followed by the release of vacuolar proteins into the cytosol, resulting in rapid cell death through the degradation of various organelles, including the nucleus [4]. Thus, vacuole destruction is one of the most important events in plant PCD, particularly during development [4].

Previous reports have shown that vacuole-processing enzymes (VPEs) located in the vacuoles are closely related to the destruction of vacuoles and play a crucial role in plant PCD [5,8,9]. VPE deficiency suppresses hypersensitive cell death in response to the tobacco mosaic virus (TMV) infection by suppressing vacuolar membranes’ disintegration in TMV-infected leaves in tobacco [8]. In addition, the features of cell death induced by fumonisin B1 (FB1), such as vacuole rupture, have been found to be completely abolished in an *Arabidopsis vpe-null* mutant lacking all four *VPE* genes [5]. Further, the caspase-1 inhibitor can inhibit MaVPE activities in susceptible banana cultivars, reduce tonoplast rupture, decrease lesion formation, and enhance stress tolerance against FocTR4 infection [9]. VPE is an endopeptidase possessing substrate-specific activity for asparagine and aspartic acid, exhibiting caspase-1-like activity (YVADase/caspase-1-like cleavage activity) in plant PCD [10]. Additionally, VPE is synthesized as an inactive protein precursor [11]. When PCD occurs in plants, the VPE precursor protein is transported to the vacuoles. Under acidic conditions, C-terminal- and N-terminal peptide precursors are sequentially excised and converted into a mature VPE model through autocatalysis [5,12]. Moreover, VPEs can cleave peptides bound to the C-terminal sides of asparagine residues and aspartic acids when exposed on the surface of proprotein precursors to generate mature proteins. Therefore, VPE is considered the initiator of the vacuolar processing system without other factors activating VPE molecules [13,14]. Plant VPEs have been separated into three subfamilies: a vegetative type (α-VPE and γ-VPE), a seed type (β-VPE), a seed coat type (δ-VPE), and an uncharacterized type [15]. The number of VPE genes in different species can be markedly different. Four genes have been described in *Arabidopsis* [5], eight in barley [16], five in rice [17], fourteen in tomato [18], seven in *Gossypium arboreum* [19], and seven in banana [9]. Plant VPEs were well known to function in regulating PCD in both developmental and defense responses widely, such as reproductive development [6], hybrid lethality [20], bud development [21], hypersensitivity reactions caused by plant pathogens [8,22], endoplasmic reticulum stress [23,24] and adversity stress [25,26]. Despite some recent progress in plant VPE genomics and expression, many studies remain to be conducted, particularly in elucidating the synthesis, transport, and action sites of VPE in plant developmental PCD due to the lack of VPE localization at the cell level.

Secretory cavities are common structural characteristics of *Citrus* plants (Rutaceae), where medicinal ingredients are synthesized and stored. Previous studies have shown that secretory cavities in *Citrus* plants are formed schizolysigenously [27,28]. PCD is involved in cell degradation during secretory cavity development [29]. Specifically, the caspase-3 like, proteasome CgPBA1 and Ca^2+^- and Zn^2+^-dependent nucleases participate in the degradation of the cell nucleus in PCD [30,31,32,33,34], whereas *CisPG21* and *CisCEL16*, which are important regulatory genes of pectinase and cellulose, participate in the degradation of the cell wall of the secretory cavity in *Citrus* fruits [28]. VPE was first reported to be associated with fruit ripening in *Citrus* fruit [35]. Additionally, we found that CgVPE1 is not only involved in PCD during TE cell development; furthermore, it may directly participate in the construction of secondary cell walls in the pericarp of *C. grandis* ‘Tomentosa’ [36]. Whether CgVPE1 is involved in PCD of the secretory cavity remains unclear. In this study, we identified a CgVPE1 protein in relation to vacuole rupture in PCD and utilized qRT-PCR, in situ hybridization, immunogold labeling location technology, and Western blotting to analyze the temporal and spatial expression characteristics of CgVPE1 during the formation of the secretory cavity by programmed cell death in fruits of *Citrus grandis* ‘Tomentosa’. Furthermore, the GST-CgVPE1 fusion protein was expressed in vitro. Additionally, combined with the changes in an intercellular pH environment in PCD, the activity of the GST-CgVPE1 protein at a different pH was investigated. These results revealed the spatiotemporal localization of CgVPE1 during the PCD process in the secretory cavity of cells, indicating that CgVPE1 could be involved in vacuole destruction, providing new information to comprehensively analyze the cytological and molecular mechanisms of secretory cavity cell PCD.

## 2. Results

### 2.1. Expression and Localization of CgVPE1 during the Development of the Secretory Cavity in C. grandis ‘Tomentosa’ Fruit

**Figure 1:**
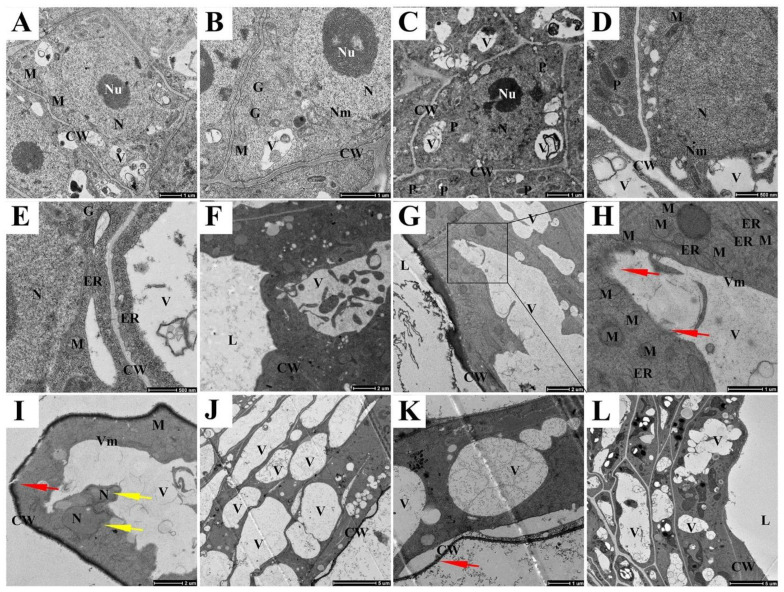
Ultrastructural changes in epithelial cells of secretory cavities during their development in *C. grandis* ‘Tomentosa’ fruit. (**A**,**B**) The early initial cell stage; (**C**–**E**) The late initial cell stage; (**F**) The lumen-forming stage; (**G**–**I**) The early lumen-expanding stage, (**H**) The enlarged image of the black box in (**G**). The yellow arrows point at nuclear region; (**J**,**K**) The middle lumen-expanding stage; Deformed cell wall in (**K**) (red arrows), and the tonoplast collapsed in (**K**); (**L**) The mature stage. Nm, nuclear membrane; Nu, nucleolus; ER, endoplasmic reticulum; V, vacuole; M, mitochondria; G, Golgi; P, plastid; L, lumen; L, J bars = 5 μm; A, F, G, I bars = 2 μm; B, C, H, K, bars = 1 μm; D, E, bars = 500 nm.

According to Bai et al. [32], combined with the morphological changes in vacuoles, the development of the secretory cavity of *C. grandis* ‘Tomentosa’ was divided into six periods: the early and late initial cell stages, the lumen-forming stage, and the early, middle lumen expanding and mature stages. At the early initial cell stage, a few small vacuoles occurred in the cytoplasm, and the nucleus occupied most of the cells. The nucleus, other organelles, and cell wall structures were intact (Figure 1A,B). At the late initial cell stage, the nucleus lost its normal shape, and its membrane became blurred and wavy (Figure 1C). More vesicles were formed (Figure 1C,D), which originated from the expanded end of the endoplasmic reticulum (Figure 1E). At the lumen-forming stage, compared to the initial cell stage, the vacuole was larger and contained more degraded substances (Figure 1F). At the early lumen-expanding stage, a large central vacuole was formed (Figure 1G), the partial vacuole membrane appeared broken and blurred (Figure 1H, red arrow), the nucleus degraded into several nuclear regions (Figure 1I, yellow arrow) and the cell walls were broken (Figure 1I, red arrow). The mitochondria and endoplasmic reticulum also became deformed (Figure 1H,I). In the middle lumen-expanding stage, the secretory cavity epithelial cells were vacuolated. The whole vacuole membrane became blurred and collapsed, and the large central vacuole contained many flocculent substances (Figure 1K). In addition, the mitochondria, endoplasmic reticulum, and other organelles disappeared. In addition, the cell walls were deformed, and plasmolysis occurred (Figure 1J,K). Finally, mature epithelial cells gradually formed, and only a few small vacuoles were distributed in functional epithelial cells (Figure 1L).

### 2.2. CgVPE1 Was a Typical Vacuolar Processing Enzyme and Had Caspase-1-like Activity

**Figure 2:**
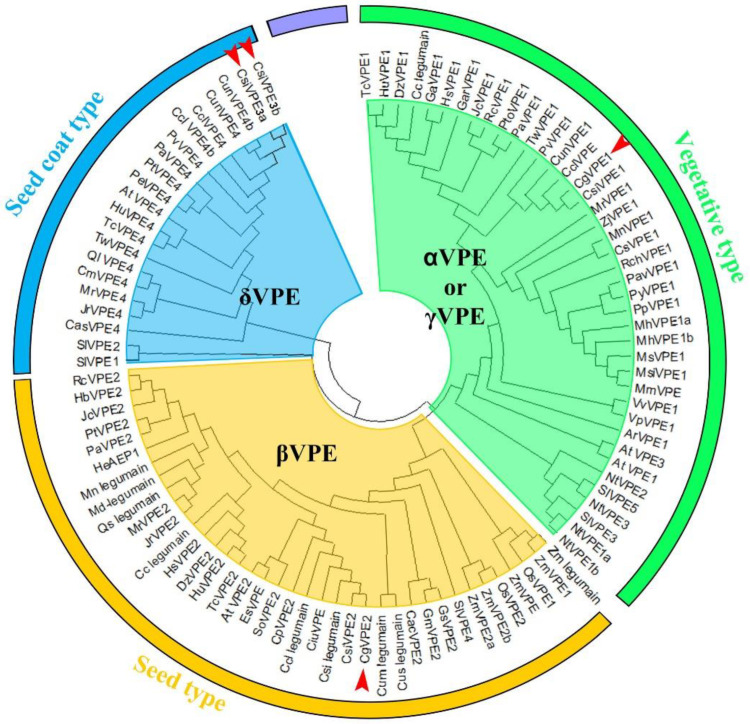
A phylogenetic tree of *Citrus grandis* VPE.

Many studies have shown that the vacuolar-processing enzyme (VPE) is related to developmental PCD [15,17,21] and is closely related to vacuole rupture in PCD [5,8,9]. The genome database showed that vacuolar processing enzyme (VPE) homologs were widely distributed in land plants, from moss (*Physcomitrella patens*) and ferns (*Ceratopteris richardii*) to seed plants [17]. Thus, we used the full-length CDs of *Arabidopsis thaliana* VPE to blast in the *Citrus sinensis* database of NCBI (https://blast.ncbi.nlm.nih.gov/Blast.cgi, (accessed on 6 July 2023)). Four genes were also identified (Table S1). We also analyzed the sequences of VPEs in *Citrus* plants to understand their function. CgVPE1 and CgVPE2 in *C. grandis* ‘Tomentosa’ and CsiVPEs in sweet orange were used for BLAST in NCBI, and nearly a hundred homologous proteins in each species were used to conduct a phylogenetic tree using MEGA-X (Figure 2). Combined with the three types of VPEs in *Arabidopsis* for analysis, the results showed that VPEs in plants are usually divided into three types: the vegetative type (such as AtαVPE and AtγVPE), seed type (such as AtβVPE) and seed coat type (such as AtδVPE). VPE1, VPE2, CsiVPE3a, and CsiVPE3b in *Citrus* belong to the vegetative, seed, and seed coat VPE, respectively. The similarities between the CgVPE1 and *Arabidopsis* AtαVPE and AtγVPE protein sequences were 71.11% and 74.01%, respectively. The number of VPE types was not completely conserved among various species. For example, there were two vegetative VPEs (AtαVPE and AtγVPE) and one seed coat type (AtδVPE) in *Arabidopsis*, while there was only one vegetative type of CgVPE1 and two seed coat types (VPE3a and VPE3b) in *Citrus* plant. We also found that CgVPE1 had a close phylogenetic relationship with *Pistacia vera* PvVPE1, as they were grouped in the same clade. We cloned *CgVPE1* from *C. grandis* ‘Tomentosa’. The full-length open reading frame (ORF) contained 1485 bp, encoding a protein with 494 amino acids (Figure S1A). The CgVPE1 sequence in *C. grandis* ‘Tomentosa’ contained peptidase C13 and legunain C domains (Figure S1B). Moreover, 1–20 aa was the signal peptide (Figure S1C).

The phylogenetic tree was analyzed using the neighbor-joining method implemented in MEGA version 7.0. Numbers at the branching points indicate the bootstrap proportions (*n* = 1000). A total of 100 VPEs were selected in NCBI, the *Citrus sinensis* database, and the TAIR database. These could be divided into three types including the vegetative (green area), seed (light yellow area), and seed coat type (blue area). The red arrows indicate CgVPE1, CgVPE2, CsiVPE3a, and CisVPE3b.

Ar = *Actinidia rufa* (ArVPE1, GFZ08559.1); At = *Arabidopsis thaliana* (AtαVPE, AT2G25940; AtβVPE, AT1G62710; AtγVPE, AT4G32940; AtδVPE, AT3G20210); Cac = *Cajanus cajan* (CacVPE2, XP_020226377.1); Cas = *Camellia sinensis* (CasVPE4, XP_028090557.1); Cc = *Corchorus capsularis* (Cclegumain, OMP02192.1; Cclegumain1, OMO66906.1); Ccl = *Citrus clementina* (Ccllegumain, XP_006440379.1; CclVPE, XP_006450795.1; CclVPE4a, XP_006450041.1; CclVPE4b, ESR63278.1); Cm = *Castanea mollissima* (CmVPE4, KAF3953759.1); Cum = *Cucumis melo* ‘makuwa’ (Cumlegumain, KAA0039681.1); Cp = *Carica papaya* (CpVPE2, XP_021901817.1); Cs = *Cannabis sativa* (CsVPE1, XP_030486503.1); Csi = *Citrus sinensis* (Csi legumain1, XP_006477253.1; CsiVPE1, NM_001288848.1; CsiVPE2, XM_006477190.4; CsiVPE3a, XM_052444192.1; CsiVPE3b, XM_052444193.1); Cun = *Citrus unshiu* (CunVPE, GAY60595.1; CunVPE1, GAY43481.1; CunVPE4a, GAY68696.1; CunVPE4b, GAY68695.1; Cuslegumain, XP_004147613.1); Dz = *Durio zibethinus* (DzVPE1, XP_022750111.1; DzVPE2, XP_022737582.1); Es = *Eutrema salsugineum* (EsβVPE2, XP_006391830.1); Gar = *Gossypium arboretum* (GarVPE1, KHG13131.1); Ga = *Gossypium austral* (GaVPE1, KAA3457979.1); Gm = *Glycine max* (GmVPE2, P49045.1); Gs = *Glycine soja* (GsVPE2, XP_028211613.1); Hb = *Hevea brasiliensis* (HbVPE2, XP_021641659.1); He = *Hybanthus enneaspermus* (HeAEP1 (asparaginyl endopeptidase 1), AWD84473.1); Hs = *Hibiscus syriacus* (HsVPE1, KAE8687616.1; HsVPE2, KAE8694577.1); Hu = *Herrania umbratical* (HuVPE1, XP_021276816.1; HuVPE2, XP_021282711.1; HuVPE4, XP_021297132.1); Jc = *Jatropha curcas* (JcVPE1, XP_012077326.1; JcVPE2, XP_012087127.1); Jr = *Juglans regia* (JrVPE2, XP_018848053.2; JrVPE4, XP_018839188.1); Md = *Malus domestica* (Md legumain, XP_008369533.1); Mh = *Malus hupehensis* ‘mengshanensis’ (MhVPE1b, ACR24644.1); Mh = *Malus hupehensis* (MhVPE1a, AGC94758.1); Mm = *Malus micromalus* (MmVPE, AGC94759.1); Mn = *Morus notabilis* (Mnlegumain, XP_010093667.1; MnVPE1, XP_010097094.1); Mr = *Morella rubra* (MrVPE1, KAB1219760.1; MrVPE2, KAB1217056.1; MrVPE4, KAB1218233.1); Ms = *Malus sikkimensis* (MsVPE1, AGC94757.1); Msi = *Malus sieversii* (MsiVPE1, AGC94756.1); Nt = *Nicotiana tabacum* (NtVPE1a; BAC54827.1; NtVPE1b, BAC54828.1; NtVPE2, BAC54829.1; NtVPE3, BAC54830.1); Os = *Oryza sativa* (OsVPE1, XP_015619322.1; OsVPE2, spB8ASK4.1); Pa = *Populus alba* (PaVPE1, XP_034891008.1; PaVPE2, XP_034897712.1; PaVPE4, XP_034909548.1); Pav = *Prunus avium* (PavVPE1, XP_021800525.1); Pe = *Populus euphratica* (PeVPE4, XP_011033074.1); Pp = *Prunus persica* (PpVPE1, XP_007222246.1); Pto = *Populus tomentosa* (PtoVPE1, ACQ91103.1); Pt = *Populus trichocarpa* (PtVPE4, XP_002310920.2; PtVPE2, XP_002304429.3); Pv = *Pistacia vera* (PvVPE1, XP_031264938.1; PvVPE4, XP_031248424.1); Py = *Prunus yedoensis* ‘nudiflora’ (PyVPE1, PQM38388.1); Ql = *Quercus lobate* (QlVPE4, XP_030922759.1); Qs = *Quercus suber* (Qs legumain, XP_023911206.1); Rc = *Ricinus communis* (RcVPE1, XP_002516472.1; RcVPE2, NP_001310660.1); Rch = *Rosa chinensis* (RchVPE1, XP_024165916.1); Sl = *Solanum lycopersicum* (SlVPE1, NP_001307953.1; SlVPE2, NP_001340697.1; SlVPE3, NP_001340698.1; SlVPE4, NP_001340699.1; SlVPE5, NP_001233955.1); So = *Syzygium oleosum* (SoVPE2, XP_030469343.1); Tc = *Theobroma cacao* (TcVPE1, EOY29855.1; TcVPE2, EOY24432.1; TcVPE4, EOY26259.1); Tw = *Tripterygium wilfordii* (TwVPE1, KAF5743206.1; TwVPE4, KAF5741619.1); Vp = *Vitis pseudoreticulata* (VpγVPE1, ALI93624.1); Vv = *Vitis vinifera* (VvVPE1, RVX17641.1); Zj = *Ziziphus jujube* (ZjVPE1, XP_015884509.1); Zm = *Zea mays* (Zm legumain, NP_001241716.1; ZmVPE, PWZ12068.1Zm VPE1b, PWZ31681.1; ZmVPE2a, NP_001105183.1; ZmVPE2b, ONM15449.1).

**Figure 3:**
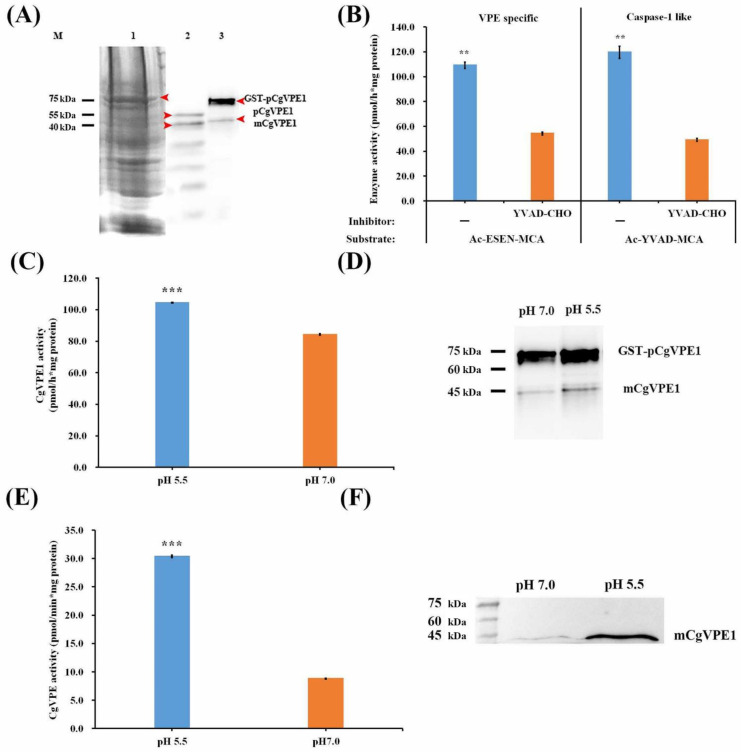
(**A**) Expression and purification of recombinant protein GST-CgVPE1. 1. SDS-PAGE analysis of total protein after ultrasonication. 2. Immunoblotting for CgVPE1 in the total protein extracted from the pericarp of *C. grandis* ‘Tomentosa’ fruits. 3. Immunoblotting for purified GST-CgVPE1 protein. The red arrows point at GST-CgVPE1, mCgVPE1 and pCgVPE1, respectively. (**B**) The enzyme assay of recombinant fusion protein GST-CgVPE1 using their substrate specificity; (**C**) The enzyme assay of recombinant fusion protein GST-CgVPE1 at different pH. (**D**) Immunoblot analysis of recombinant fusion protein GST-CgVPE1 at different pH. (**E**) The enzyme assay of the total protein extracted from the pericarp of *Citrus grandis* ‘Tomentosa’ fruits at different pH. (**F**) Immunoblot analysis of total protein extracted from the pericarp of *Citrus grandis* ‘Tomentosa’ fruits at different pH. “**”, “***” indicate a significant difference at *p* ≤ 0.01 and *p* ≤ 0.001, respectively. (*t*-test). mCgVPE1, mature CgVPE1 protein; pCgVPE1, proenzyme of CgVPE1.

In plants, VPE proteins are usually synthesized as proenzymes that become mature proteases through self-cleavage [37]. We performed immunoblotting for CgVPE1 in the proteins of *C. grandis* ‘Tomentosa’ fruits to evaluate the CgVPE1 antibody’s specificity. About 54 kDa CgVPE1 proenzymes and about 40 kDa mature enzymes were detected by Western blotting (Figure S2C and Figure 3A). To further explore the characteristics of CgVPE1 in *Citrus* plants, we constructed an expression vector, *pGEX-4T1-CgVPE1,* and expressed it in *E. Coli* (DE3). The recombinant fusion protein GST-CgVPE1 was purified. Many recombinant proteins (GST-pCgVPE1) of approximately 75–80 kDa and a few mature CgVPE1 proteins (mCgVPE1) of approximately 40 kDa were obtained (Figure 3A). We used a specific substrate of VPE (Ac-ESEN-MCA) and a specific substrate of caspase-1 (Ac-YVAD-MCA) to determine whether the recombinant GST-CgVPE1 fusion protein had both VPE activity and caspase-1-like activity at pH 5.5. The caspase-1 inhibitor (Ac-YVAD-CHO) inhibited VPE and caspase-1-like activities (Figure 3B). We also found that the VPE activity of GST-CgVPE1 at an acidic pH of 5.5 was significantly higher than that at a normal pH (7.0) (Figure 3C). Moreover, the same amount of GST-pCgVPE1 was transformed into more mature CgVPE1 (mCgVPE1) at pH 5.5, compared to that at pH 7.0, using immunoblot analysis with CgVPE1-specific antibodies (Figure 3D). In addition, when the equal total protein extracted from the pericarp of *C. grandis* ‘Tomentosa’ fruits was reacted in a neutral pH 7.0 buffer and acidic pH 5.5 buffer, respectively, we only detected the mature CgVPE1 proteins and more mCgVPE1 were detected in acidic buffers of 5.5 (Figure 3F). They also exhibited higher VPE activity at pH 5.5 (Figure 3E). Taken together, CgVPE1 could be transformed into a mature protein (mCgVPE1) at pH 5.5 through self-cleavage, which showed higher VPE activity in an acidic environment.

### 2.3. Expression and Localization of CgVPE1 during the Development of the Secretory Cavity in C. grandis ‘Tomentosa’ Fruits

**Figure 4:**
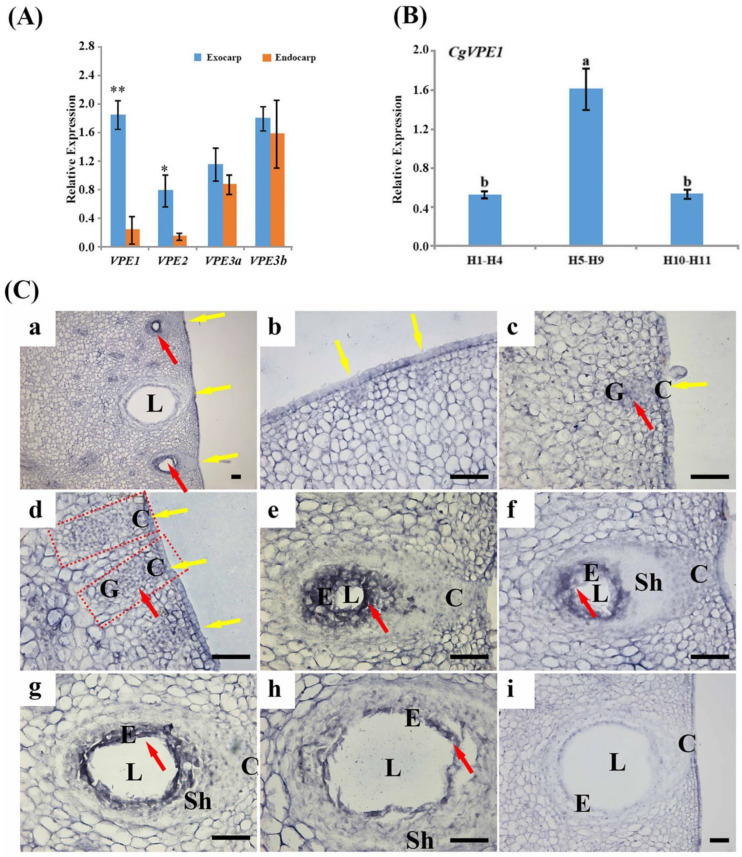
*CgVPE1* expression pattern during the development of secretory cavities in *C. grandis* ‘Tomentosa’ fruit. (**A**) The expression of *VPEs* in the exocarp and endocarp of *C. grandis* ‘Tomentosa’ fruits. “*”, “**” indicate a significant difference at *p* ≤ 0.05 and *p* ≤ 0.01, respectively (*t*-test); (**B**) The expression of *CgVPE1* in different developmental stages of *C. grandis* ‘Tomentosa’ fruit. Different letters indicate significant differences at *p* ≤ 0.05 (Duncan’s multiple comparisons); (**C**) *CgVPE1* expression pattern: (**a**) *CgVPE1* expression pattern in the pericarp of *C. grandis* ‘Tomentosa’; (**b**) The early initial cell stage; (**c**) The middle initial cell stage; (**d**) The late initial cell stage (the red dot box); (**e**) The lumen-forming stage; (**f**) The early lumen-expanding stage; (**g**,**h**) The middle lumen-expanding stage; (**i**) The late lumen-expanding stage. The yellow arrows point at the secretory cavity, and the red arrow indicates the expression signals of *CgVPE1*. E, epithelial cells; L, lumen; Sh, sheath cell; C, conical part cells; G, the globular part; bars = 50 µm.

qRT-PCR was used to analyze the expression levels of VPEs. Since the same pericarp contained secretory cavities of different developmental stages with different morphological characteristics, the proportion of different developmental stages of *C. grandis* ‘Tomentosa’ secretory cavities in the ovary or young fruit at different sizes was performed by paraffin section and microscopic observation (Figure S3). We divided the pericarps of *C. grandis* ‘Tomentosa’ fruit at different growth stages (H1–H11) into three mixed samples (H1–H4, H5–H9, and H10–H11) according to the morphology of the vacuoles. Of the secretory cavities, 89.75% were at the initial cell stage (H1–H4), 64.2% were in both the lumen-forming and early lumen-expanding stage (H5–H9), and 63.5% were in the late lumen-expanding stage (H10–H11) in the exocarp of *C. grandis* ‘Tomentosa’ fruit. First, we analyzed the expression of VPEs between the exocarp containing secretory cavities and the endocarp without the secretory cavity of the young fruit (H7–H9, most of the secretory cavities were in the lumen-forming and early lumen-expanding stages) to screen out VPEs in relation to the development of secretory cavities (Figure 4A). The expression of *CgVPE1* and *CgVPE2* in the exocarp was significantly higher than that in the endocarp, and the difference in *CgVPE1* expression levels was highly significant (*p* < 0.01). To further verify the function of CgVPE1, we analyzed the different expressions of *CgVPE1* during the development of secretory cavities in *C. grandis* ‘Tomentosa’ fruits (Figure 4B). The results showed that the *CgVPE1* expression level increased first and then decreased during the developmental process of the exocarp of *C. grandis* ‘Tomentosa’ fruits. *CgVPE1* expression levels in the peel during the H5–H9 stage were significantly higher than those during the H1–H4 and H10–H11 stages. In situ hybridization was performed to determine the spatial and temporal expression patterns of *CgVPE1* during secretory cavity development (Figure 4C). The *CgVPE1*-specific RNA fragment was designed as an antisense probe, and a sense probe was used as a control. Compared to the negative control (Figure S4B,C), the *CgVPE1* expression signal appeared blue and bluish-violet (Figure 4C). Expectedly, *CgVPE1* was specifically expressed in the developing epithelial cells of the secretory cavity in *C. grandis* ‘Tomentosa’ fruits (Figure 4Ca). The *CgVPE1* expression was detected in the middle initial cell stage of the secretory cavities. The transcripts of *CgVPE1* increased continuously from the middle initial cell stage to the lumen-forming stage (Figure 4Cc–e). Then, *CgVPE1* transcripts decreased from the early lumen-expanding stage to the late lumen-expanding stage (Figure 4Cf–i). At the late lumen-expanding stage, almost no signal was observed in the epithelial cells (Figure 4Ci). Thus, the specific *CgVPE1* expression was consistent with qRT-PCR data during secretory cavity development. Additionally, the *CgVPE1* signal was clearly observed in vascular bundle cells, presumably in relation to the formation of tracheary elements (Figure S4Aj, [36]).

To explore the possible function of CgVPE1 in the PCD during secretory cavity development, we used immunofluorescence to determine the spatial and temporal distribution of CgVPE1 during secretory cavity development (Figure S5), which was consistent with CgVPE1 expression levels determined by in situ hybridization. Green fluorescence was specifically concentrated in the secretory cavity. The green fluorescence of CgVPE1 was strongest during the lumen-forming stage. However, a weaker fluorescence signal of CgVPE1 was observed during the late initial cell stage (Figure S5A–D) and the middle lumen-expanding stage (Figure S5I–L). During the middle lumen-expanding stage, CgVPE1 signals in the innermost secretory cavity cell layer around the lumen almost disappeared (Figure S5E–H), which could be related to cell destruction.

**Figure 5:**
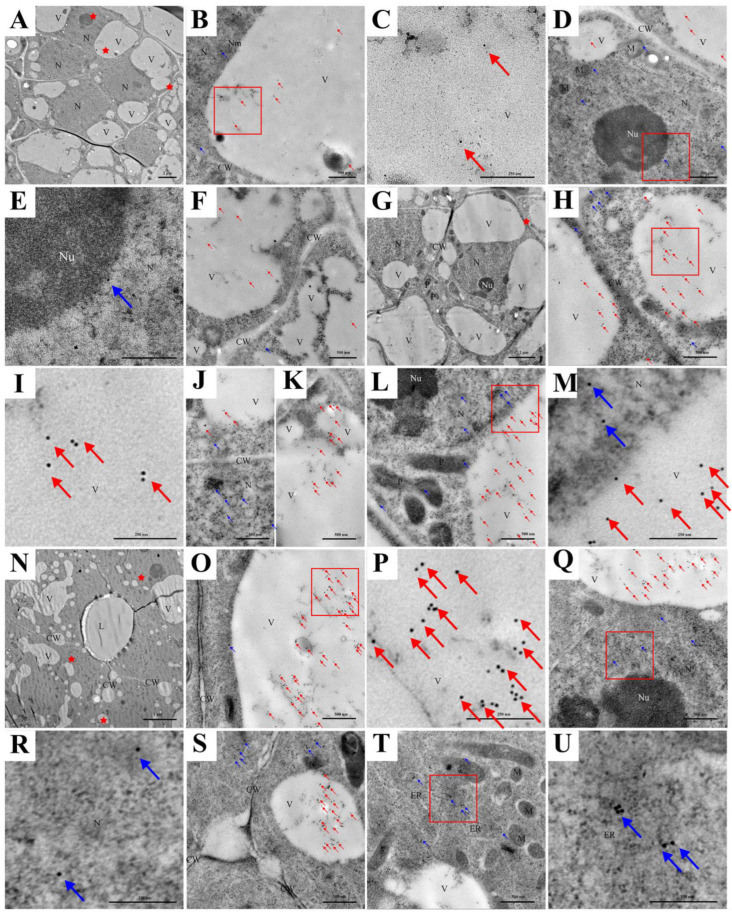
Subcellular distribution analysis of CgVPE1. (**A**–**F**) The early initial cell stage. (**B**,**D**,**F**) are the enlarged image of the red stars area in (**A**), respectively. (**C**,**E**) are the magnified images of the red boxes in (**B**,**D**), respectively. (**G**–**M**) The late initial cell stage. (**H**) is the enlarged image of the red stars area in (**G**). (**I**,**M**) are the magnified images of the red boxes in (**H**,**L**), respectively. (**N**–**U**) The lumen-forming stage. (**Q**,**S**,**T**) are the enlarged image of the red stars area in (**N**), respectively. (**P**,**R**,**U**) are the magnified images of the red boxes in (**O**,**Q**,**T**), respectively; red arrows point at immunogold particles in the vacuole, and blue arrows point at immunogold particles in another position except for the vacuole. L, lumen; V, vacuole; CW, cell wall; N, nucleus; Nu, nucleolus; M, mitochondria; P, plastids; (**A**,**G**) bars = 2 μm; (**J**), bars = 5 μm; (**B**,**D**,**F**,**H**,**J**–**L**,**O**,**P**,**S**,**T**) bars = 500 nm; (**C**,**E**,**I**,**M**,**P**,**R**,**U**) bars= 250 nm.

**Figure 6:**
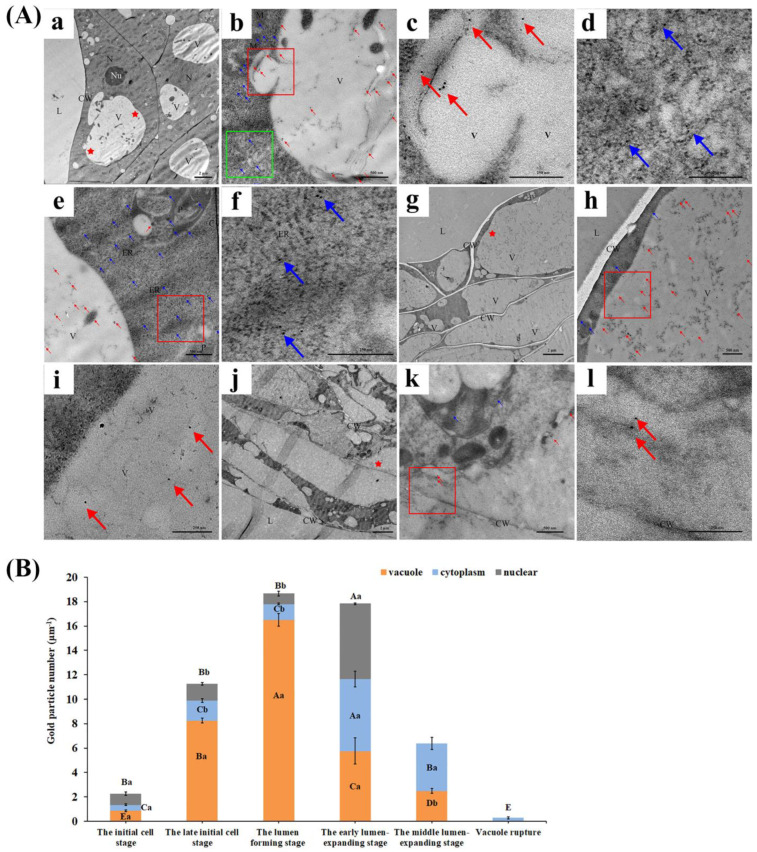
(**A**) Subcellular distribution analysis of CgVPE1. (**a**–**f**) The early lumen-expanding stage. (**b**,**e**) are the enlarged image of the red stars area in (**a**), respectively; (**c**,**d**) is the magnified images of the red box and green box in (**b**), respectively. (**f**) is the magnified image of the red box in (**e**). (**g**–**i**) The middle lumen-expanding stage. (**h**) is the enlarged image of the red star area in (**g**). (**i**) is the magnified image of the red box in (**h**); (**j**–**l**) The epithelial cells are destroyed, (**k**) is the enlarged image of the red stars area in (**j**). (**l**) is the magnified image of the red box in (**k**); red arrows point at immunogold particles in the vacuole, and blue arrows point at immunogold particles in another position except for the vacuole. L, lumen; V, vacuole; CW, cell wall; N, nucleus; Nu, nucleolus; M, mitochondria; P, plastids; (**a**,**g**,**i**) bars = 2 μm; (**b**,**e**,**h**,**k**) bars = 500 nm; (**c**,**d**,**f**,**i**,**l**) bars= 250 nm. (**B**) Statistics of the number of gold particles per unit area in different structures at different developmental stages of the secretory cavity in *C. grandis* ‘Tomentosa’ fruit. Different lowercase letters indicate significant differences in the number of gold particles in different structures at the same developmental stage, *p* ≤ 0.05. Different capital letters indicate significant differences in the number of gold particles in the same structures at different developmental stages, *p* ≤ 0.05, Duncan’s multiple comparisons.

In addition, immunocytochemical localization was used to determine the subcellular localization of CgVPE1 in the secretory cavity cells at different developmental stages (Figure 5 and Figure 6). ImageJ v1.8.0.112 software was used to count the number of gold particles in the secretory cavity cells (Figure 6B). The results showed that the CgVPE1 protein location exhibited temporal and spatial expression specificity with vacuole morphology changes during the development of secretory cavity cells. At the early initial cell stage, a few CgVPE1 proteins were located in the vacuoles and nuclei of secretory cavity cells; however, there was no significant difference in their amounts (Figure 5A–E and Figure 6B). Additionally, there was no significant difference in the content of the CgVPE1 protein in the vacuoles between secretory cavity cells and ordinary parenchyma cells (Figure S6A). Almost no CgVPE1 was distributed in the mitochondria, plastids, or cell walls (Figure 5D,F). At the late initial cell stage, the CgVPE1 protein was concentrated in the vacuoles, and its amount increased significantly (Figure 5G–I and Figure 6B). Interestingly, the CgVPE1 protein appeared in vesicles and vacuoles, and some vesicles containing the CgVPE1 protein were found to fuse with vacuoles (Figure 5J,K). The number of CgVPE1 in the nucleus was not significantly different from that in the early initial cell stage (Figure 5L,M and Figure 6B). At the lumen-forming stage (Figure 5N), it is worth noting that there was a large amount of CgVPE1 protein in the vacuoles of epithelial cells, which reached a maximum in the vacuoles (Figure 5O–Q,S and Figure 6B). However, the number of CgVPE1 in the nucleus remained unchanged (Figure 5R). No CgVPE1 was observed in the cell wall (Figure 5S). We also observed that a few CgVPE1 accumulated in the endoplasmic reticulum (Figure 5T,U). In the early lumen-expanding stage (Figure 6A), the CgVPE1 content in the vacuole was significantly reduced compared to that in the lumen-forming stage (Figure 6Aa–c,B). However, the CgVPE1 content in the cytoplasm and nucleus significantly increased (Figure 6B), which could be related to the release of CgVPE1 into the cytoplasm from the vacuole after its partial rupture. A few CgVPE1 proteins were distributed in the plastids and endoplasm reticulum (Figure 6Ad–f). At the middle lumen-expanding stage, there were still a few CgVPE1 proteins in both the vacuole and cytoplasm; however, there was no significant difference in their levels (Figure 6Ag–i,B). With the rapid enlargement of the secretory cavity, some epithelial cells began to collapse (Figure 6Aj–l). The cytoplasmic matrix dissolved in the vacuole, and the cytoplasm loosened and separated from the cell wall. At the time, CgVPE1 proteins in the cell decreased sharply, and only a few CgVPE1 proteins remained in the cytoplasm, destroying the vacuoles (Figure 6Ak,l). In the negative control (the secondary antibody was replaced with PBS, or the primary antibody was replaced with PBS), no CgVPE1 proteins were observed in the vacuoles, cytoplasm, or organelles (Figure S6B,C). According to the statistics of the number of gold particles per unit area at different developmental stages of the secretory cavity in *C. grandis* ‘Tomentosa’ fruit, we found that the CgVPE1 content in both vacuoles and whole cells initially increased and then decreased. CgVPE1 proteins were mainly concentrated in the vacuole before the early lumen expansion stage. After the early lumen expansion stage, the content of CgVPE1 decreased sharply. CgVPE1 was mainly located in the cytoplasm (Figure 6B). To further confirm the results of the immunogold particles’ localization of CgVPE1 into the secretory cavities of *C. grandis* ‘Tomentosa’, the online tool (https://wolfpsort.hgc.jp/ (accessed on 20 May 2022)) was used to predict the subcellular location of the CgVPE1 protein (Table S2). The results showed that CgVPE1 was most likely localized in vacuoles, followed by chloroplasts, cell nuclei, mitochondria, plastids, endoplasmic reticulum, and Golgi bodies, which have certain possibilities. It could be determined that CgVPE1 should be primarily distributed in vacuoles of the secretory cavity cells of *C. grandis* ‘Tomentosa’ fruits; however, its distribution in other organelles or nuclei cannot be ruled out.

**Figure 7:**
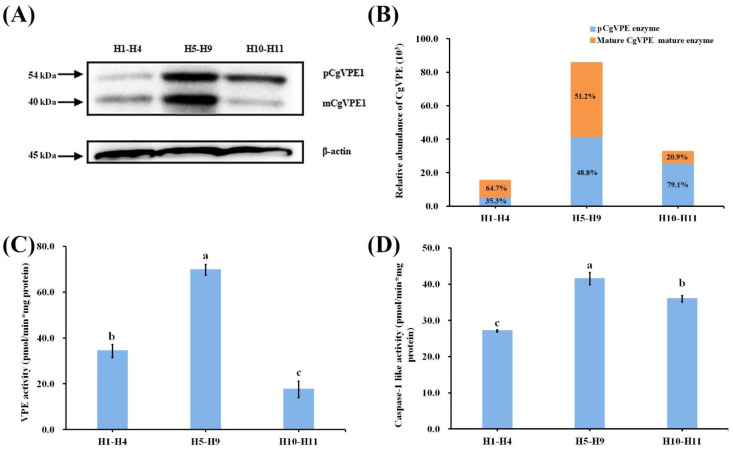
(**A**) Immunoblot analysis of total pericarp protein extracts from H1 to H11 with an anti-CgVPE1 body. (**B**) Relative abundance of CgVPE1 during the secretory cavity development. (**C**,**D**) Enzyme assay of total protein extracted from pericarp of *C. grandis* ‘Tomentosa’ fruits. Different letters indicate a significant difference at *p* ≤ 0.05, Duncan’s multiple comparisons.

Given that the VPE protein can self-catalyze activation from the proenzyme to the mature enzyme, we performed immunoblotting for CgVPE1 in the mixed pericarp at three stages (H1–H4, H5–H9, and H10–H11) to evaluate CgVPE1 maturation using an anti-CgVPE1 body (Figure 7A,B). These results suggest that the CgVPE1 content first increased and then decreased from H1 to H11. The content of the 40 kDa mature enzyme (mCgVPE1) was higher than that of the 54 kDa proenzyme (pCgVPE1) from H1 to H4, which could be related to CgVPE1 located in the vacuole of the secretory cavity cell at the initial cell stage. Both the proenzyme and mature enzyme contents increased from H5 to H9. The relative content of the proenzyme was higher than that of the mature enzyme from H10 to H11, which could be related to the collapse of the vacuolar membrane (Figure 7A,B). Subsequently, we determined the activities of VPE and caspase-1 during secretory cavity development in the pericarp of *Citrus grandis* ‘Tomentosa’ fruits. The activity of VPE first increased and then decreased, which was consistent with the change in the protein content of CgVPE1 (Figure 7C). Caspase-1 activity showed similar results; however, it remained at a high activity at the H10-H11 stage, which indicated that some other proteases with caspase-1 activity existed (Figure 7D).

## 3. Discussion

### 3.1. CgVPE1 Was a Vegetative Type with VPE and Caspase-1-like Activities

PCD plant proteases contain many proteases that are divided into different families and subfamilies based on their evolutionary and functional relationships. Legumain-like proteases, also called vacuolar processing enzymes (VPEs), play multifunctional roles in the development and cell death of different plant organs [15]. VPE was reported to catalyze the caspase-1 substrate (N-acetyl-YVAD-MCA) in tobacco [38], first revealing the existence of caspase-like proteases associated with plant cell death. VPE, a cysteine protease also known as asparaginyl endopeptidase (AEP) or legumain, is located in vacuoles and is widely involved in plant PCD, including growth, development [21] and stress-induced PCD [25,39,40]. There are four VPEs (αVPE, βVPE, γVPE, and δVPE) in the Arabidopsis genome, which can be divided into three types: vegetative types (αVPE and γVPE) [41,42,43], seed types (βVPE) [41,44] and seed coat type (δVPE) [6]. We screened two candidate VPE genes (CgVPE1 and CgVPE2) that could regulate programmed cell death during the development of secretory cavities. Because the expression level of CgVPE1 exhibited a highly significant difference between the exocarp with secretory cavities and the endocarp without secretory cavities, CgVPE1 was selected as a candidate protein in relation to PCD. Additionally, phylogenetic analysis showed that CgVPE1 had a high similarity to AtγVPE and AtαVPE and was classified as a vegetative type.

In a previous study, VPE had a specific substrate activity toward asparagine or aspartic acid residues. They had similar structural and enzymatic properties to caspase-1 in animals exhibiting caspase-1-like/YVADase cleavage activity [11]. In addition, we found the recombinant GST-CgVPE1 fusion protein in vitro to exhibit VPE and caspase-1-like activity. Thus, we concluded that CgVPE1 is a vegetative VPE that possesses both VPE activity and caspase-1-like activity.

### 3.2. CgVPE1 Plays a Crucial Role in Tonoplast Collapse during the Developmental PCD of Secretory Cavity Cells in C. grandis ‘Tomentosa’ Fruits

PCD is a relatively conserved basic physiological process that is involved in developing various animal and plant adversities [45,46]. During apoptosis, dead cells are engulfed by macrophages. However, the vacuolar system also plays an important role in plant PCD [47]. The destruction of the vacuolar membrane is considered a key event in certain plant PCD [20,48], such as the hypersensitivity response (HR), which is caused by plant pathogens [5] and the formation of tracheary elements (TEs) [7]. The vacuole membrane disintegrates before cell death, and the continuous rupture of the vacuole membrane results in the final collapse of the vacuole and plasmolysis [8]. Vacuolar hydrolytic enzymes escape the vacuole, enter the cytosol, and degrade cellular components [11], destroying the entire cell, including the cell wall [27]. Notably, our results showed that the nuclear, endoplasmic reticulum, mitochondria, and even cell walls gradually degraded or deformed after the local destruction of the vacuole membrane. Finally, these organelles disappeared after the collapse of the vacuole during the secretory cavity development in *C. grandis* ‘Tomentosa’ fruits, which has also been demonstrated in previous studies [29,30]. Taken together, we speculate that the destruction of the vacuole membrane is an important event for the PCD of secretory cavity cells in *C. grandis* ‘Tomentosa’ fruits.

VPE, located in the vacuole, is widely involved in plant developmental PCD [17,47] and is thought to be related to vacuole rupture [5,8]. Hatsugai et al. used a virus-induced gene silencing (VIGS) strategy to provide evidence that VPE with caspase-1-like activity is essential for TMV-induced hypersensitive cell death by controlling vacuolar rupture in *N. benthamiana*. In contrast, VPE-silenced plants do not undergo vacuolar membrane disintegration or cell death [8]. Mino et al. found that VPE could be involved in the structural anomalies of the tonoplast, which lead to cell death triggered by vacuolar collapse in hybrid seedlings [20]. Here, we identified a VPE named CgVPE1, which is expressed particularly in secretory cavity cells during secretory cavity development. Additionally, the expression level of CgVPE1 reached peaks before vacuole rupture. Correspondingly, the immunocytochemical localization of CgVPE1 showed that abundant CgVPE1 proteins were concentrated in the vacuole before tonoplast rupture. Further immunoblotting and enzyme activity analyses showed that high CgVPE1 expression and high VPE or caspase-1-like activities were closely related to vacuole collapse during PCD. Taken together, these results suggest that CgVPE1 could play a crucial role in the PCD of secretory cavity cells, especially in vacuole rupture and in *C. grandis* ‘Tomentosa’ fruits.

To investigate the cytological mechanism of VPEs when acting on vacuolar destruction in the PCD of *Citrus* plants, we used an immunocytochemical technique to track the spatiotemporal localization of CgVPE1 during the development of PCD in secretory cavity cells. Based on the changes in CgVPE1 localization, number, and distribution, CgVPE1 was first synthesized on the rough endoplasmic reticulum and then transported into the vacuole by small vesicles fusing with vacuoles. These results are consistent with a previous cytobiological hypothesis [11]. In the early stage of PCD, large amounts of the CgVPE1 protein were found in vacuoles. We detected two bands of about 54 kDa and 40 kDa in size in the total protein extracted from the pericarp of *C. grandis* ‘Tomentosa’, which were the proenzyme of CgVPE1 (pCgVPE1) and mature enzyme of CgVPE1 (mCgVPE1), respectively. Notably, these changes in the two types of VPE proteins from pCgVPE1 to mCgVPE1 were closely related to changes in CgVPE1 localization during secretory cavity development. When the content of CgVPE1 labeled with immune colloidal gold particles in the vacuoles was higher than that in the cytoplasm, a higher content of mature CgVPE1 proteins (mCgVPE1) was detected by immunoblotting. Biochemical experiments have shown that the enzymatic latency of plant VPE zymogens is conferred by the C-terminal pro-domain, which has been reported to become (auto-) proteolytically cleaved at an acidic pH for conversion into an active form [7,12,49]. The vacuoles are in an acidic environment (pH ≈ 4.0–5.5) which could provide a place of a self-shear activation site for VPE [50]. The self-catalytic conversion of inactive precursor proteins into functional VPE resembled the processing and activation of caspase 1 [5,12,51]. Correspondingly, we found that more pro-CgVPE1 proteins (about 54 kDa) or GST-CgVPE1 (about 75 kDa) were converted into mature CgVPE proteins (about 40 kDa) at acidic pH5.5 through the recombinant protein or total protein from *C. grandis* ‘Tomentosa’ fruits. In conclusion, pro-CgVPE1 can be self-cleaved and activated into the mature protein (mCgVPE1) in vacuoles. Yamada et al. reported that VPEs mainly affect the maturation and activation of vacuolar proteins [52]. VPE activity directly affects the activity of its target proteins to regulate vacuole function (reviewed by Yamada et al.) [52]. βVPE in *Arabidopsis* is directly involved in activating cysteine protease (CEP1) before the vacuole is destroyed, indirectly affecting pollen development and tapetal cell degradation. In vitro recombination experiments have also proved that the βVPE protein self-cleaves into the 27 kDa mature protein at pH 5.2 and then activates the CEP1 protein [53]. Similarly, γVPE can also affect the PCD of *Arabidopsis* xylem fiber cells by activating the CEP1 protein [54]. These reports demonstrate that VPE is involved in the destruction of vacuoles as an activated hydrolase protein based on molecular biology, genetics, and biochemistry. Using immunocytochemistry and electron microscopy, we found that the mass accumulation of CgVPE1 occurred before the vacuole collapsed, and the gradual disintegration of organelles and cytoplasm was observed after vacuole collapse during the PCD of secretory cavity cells. Taken together, we speculated that CgVPE1 could act as an activator of the vacuolar hydrolyzed protein and participate in vacuolar destruction during the formation of secretory cavities by PCD in the fruit of *C. grandis* ‘Tomentosa’.

**Figure 8:**
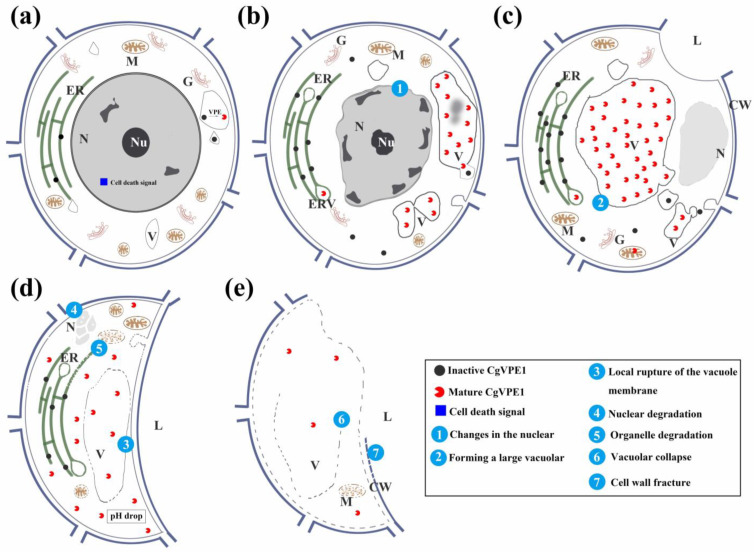
The mechanism of vacuole destruction during the PCD of epithelial cells in the secretory cavity of *C. grandis* ‘Tomentosa’ fruits. (**a**) In the early initial cell stage, cell morphology was normal, and there was very little CgVPE1 in the vacuole before PCD began; (**b**) In the late initial cell stage, the cell nucleus began to show significant degradation characteristics. The CgVPE1 in the vacuole gradually increased in preparation for activating hydrolytic protease to destroy the vacuoles; (**c**) In the lumen forming stage, the most important change in the cell was that the cell wall began to expand to form an interspace, and the cell wall was separated to form a lumen. Additionally, CgVPE1 accumulated in the vacuole to reach a maximum; (**d**) In the early lumen expanding stage, the vacuole began to rupture locally to make the cytoplasmic pH decrease. Various hydrolases and CgVPE1 were activated; however, the CgVPE1 content in the cell began to decrease; mitochondria, endoplasmic reticulum, and other organelles began to degrade; (**e**) The vacuole ruptured, the plasma membrane degraded, and the cell wall was broken. Finally, the cell died, and the CgVPE1 in the cell almost disappeared. N, nucleus; Nu, nucleolus; M, mitochondria; ER, endoplasmic reticulum; G, Golgi apparatus; CW, cell wall; L, lumen; ERV, swollen endoplasmic reticulum vesicle; V, vacuole.

In conclusion, a working model diagram of the synthesis, transportation, and regulation of CgVPE1 during the development of the secretory cavity in *C. grandis* ‘Tomentosa’ fruits was proposed in combination with the work in [55] (Figure 8). When the PCD was initiated during the development of the secretory cavities in *C. grandis* ‘Tomentosa’, a mass of the pro-CgVPE1 protein was synthesized on the endoplasmic reticulum and transported to the vacuoles through swollen vesicles from the endoplasmic reticulum. In the vacuole, pro-CgVPE1 was self-cleaved and activated to mature CgVPE1 (mCgVPE1) in an acidic environment. It then activated other hydrolyzed proteins involved in PCD. Subsequently, a tonoplast lesion formed, and the entire vacuole collapsed. Finally, vacuolar hydrolytic enzymes such as aspartate proteinases, cysteine proteinases, nucleases, pectinase, and cellulose [27,50,56] were released into the cytoplasm to participate in the degradation of organelles, cytoplasm, and cell walls, resulting in cell rupture.

## 4. Materials and Methods

### 4.1. Experimental Materials

Flowers and young fruits of *C. grandis* ‘Tomentosa’ were obtained from a 15-year-old tree at a farm of the South China Agricultural University, Guangzhou, China (23°10′2″ N, 113°21′53″ E). We divided the ovaries and young fruits into 11 growth stages according to their morphological size at different growth stages (Figure S7A,B).

### 4.2. Experimental Method

#### 4.2.1. Transmission Electron Microscopy (TEM)

Small blocks of exocarp and the ovaries (1 × 1 × 2 mm) containing different developmental secretory cavities were fixed in 2% glutaraldehyde and 3% paraformaldehyde with a phosphate-buffered solution (PBS, pH 7.2) overnight at 4 °C and were then washed with PBS three times. The samples were post-fixed in 1% osmium for 2 h before washing three times with PBS at room temperature. The samples were then passed through a series of alcohol solutions, dehydrated, and embedded in Epon 812 (SPI Supplies, West Chester, PA, USA). The samples were cut into 1–2 µm thick sections using a Leica RM2155 microtome (Leica, Weztlar, Germany). Based on these thin sections, the samples were cut into 60–90 nm thick sections using a Leica EM UC7 ultramicrotome (Leica, Weztlar, Germany). Sections in the copper grid were stained and observed as described by Bai et al. [32].

#### 4.2.2. qRT-PCR Analyses

The total RNA from the ovary walls and fruit exocarps containing different developmental secretory cavities and the endocarp without secretory cavities was extracted using the Column Plant RNAout 2.0 kit (TIANDZ, Beijing, China), according to the manufacturer’s protocol. First-strand complementary DNA (cDNA) was generated using a PrimeScript 1st Strand cDNA Synthesis Kit (TaKaRa, Beijing, China). Quantitative primers for the target and reference genes were designed using Primer Premier 5 (Table S3). The qRT-PCR analysis of the *CgVPE1* gene was performed using the 2× SYBR Green qPCR mix (Bio-Rad, USA). The qRT-PCR conditions were as follows: 95 °C for 30 s, 40 cycles at 95 °C for 5 s, and 60 °C for 30 s. The relative expression levels of genes were calculated using the 2^−ΔΔCt^ method [57]. Three biological replicates and three technical replicates were used in each experiment.

#### 4.2.3. Cloning and Sequence Analysis of CgVPE1 cDNAs

The total RNA was extracted as described previously. The primers used for cloning are listed in Table S3. The coding sequence of *CgVPE1* (Cs4g18790) was subcloned into the pMD™19-T vector using the pMD™19-T Vector Cloning Kit (TaKaRa, Beijing, China) and was sequenced by Sangon Biotech (Guangzhou, China). The 1458 bp cDNA sequence was obtained and translated into an amino acid sequence using DNAMAN 8.0 (Lynnon Biosoft, San Ramon, CA, USA). The phylogenetic analysis of CgVPE1 was conducted using MEGA-X v1.2.6 software and the neighbor-joining method.

#### 4.2.4. In Situ Hybridization (ISH)

Tissues were fixed and hybridized according to Bai et al. [32] with the following modifications. A 216 bp *CgVPE1* cDNA segment was used to synthesize the antisense and sense probes (Table S4). RNA was hybridized, and hybridized probes were detected according to the protocol described by Bai et al. [32]. Slides were observed and photographed using a Leica DM 6 B microscope (Leica, Weztlar, Germany).

#### 4.2.5. Western Blot

Mixed samples (H1–H4, H5–H9, and H10–H11) of the ovary walls and fruit exocarps were used to extract the total protein using a Microextraction Kit for the Total Plant Protein (TIANDZ, Beijing, China). Equal amounts of this protein from different mixed samples were separated using 12% (*w*/*v*) sodium dodecyl sulfate and were then transferred onto PDVF membranes (BioRad, Hercules, CA, USA) (30 V, 12 h). Then, the membrane was incubated in the purified anti-CgVPE1 antibodies, which were diluted to 1:3000 with a PBST (10 mM PBS, 0.15 M NaCl, 0.02% Tween-20 at pH 7.2) buffer for 12 h at 4 °C. Actin was used as the reference protein. The membrane was then washed with PBST three times for 10 min and incubated with secondary antibodies (Goat Anti-Rabbit IgG, Polyclonal, Wuhan ABclonal Biological Technology Co., Ltd., Wuhan, China) before being diluted to 1:10,000 for 2 h at room temperature and washed five times for 6 min each. This was followed by color development using horseradish peroxidase (HRP)-enhanced chemiluminescence (ECL) assays.

A 360 bp fragment encoding *CgVPE1* was subcloned into the pET-28a vector and expressed in *Escherichia coli* Rosetta (DE3). The expression, extraction, purification, and in vivo immunization of CgVPE1 were performed according to the procedures described by Bai et al. [32]. The immunogen protein was approximately 35 kDa mixed with Freund’s adjuvant into two Japanese big-ear rabbits for in vivo immunization. This experiment was performed at the Wuhan ABclonal Biological Technology Co., Ltd. (Wuhan, China). The rabbits were injected four times over 60 d. The collection and detection of anti-CgVPE1 was performed according to Bai et al. [32]. Anti-CgVPE1 dilution (1:1000) could detect endogenous proteins (from in vitro expression) and exogenous proteins (from the fruit exocarps of *C. grandis* ‘Tomentosa’) (Figure S2A–C).

#### 4.2.6. Immunofluorescence Localization of CgVPE1

The samples were fixed in 4% paraformaldehyde for 16 h at 4 °C. Paraffin-embedded sections were prepared as previously described by Liang et al. [34]. Immunofluorescence signals of the CgVPE1 protein were determined according to the manufacturer’s instructions (Boster Biotechnology Co., Ltd., Wuhan, China). The primary antibody (anti-CgVPE1 body) was diluted with phosphate-buffered saline (PBS, 1:100) and incubated at 37 °C for 1 h.

#### 4.2.7. Immunocytochemical Localization

The exocarp and ovary walls (1 × 1 × 2 mm) containing secretory cavities at different growth stages were fixed and embedded, as described by Bai et al. [32]. The sections on the nickel grid were then labeled with a purified CgVPE1 antibody (1:20) in PBST (10 mM PBS, 0.15 M NaCl, 0.02% Tween-20, 0.02% NaN3 at pH 7.2). The negative control was not labeled with the purified CgVPE1 antibody. After five washes with PBST, the sections were labeled with colloidal gold particles (10 nm) and coupled to goat anti-rabbit immunoglobulin G (1:50) in PBST. These sections were examined and photographed using a Philips Fei-Tecnai 12 TEM. The ImageJ software was used to count the number of gold particles in the secretory cavity cells.

#### 4.2.8. Expression and Purification of Recombinant Protein

The open reading frame (ORF) of *CgVPE1* was amplified by a PCR using two primers inserted into pGEX-4T1. The primers used are listed in Supplementary Table S3. The *pGEX-4T1/CgVPE1* plasmid was transformed into *E. coli* Rosetta (DE3) cells. *E. coli* Rosetta (DE3) cells containing the GST-CgVPE1 constructs were induced with 0.1 mM isopropyl β-D-1-thiogalactopyranoside (IPTG) for 18 h (16 °C, 120 rpm). GST-CgVPE1 fusion proteins were predominantly expressed in inclusion bodies. Therefore, we increased the volume of the expressed bacteria to 5 L. The recombinant GST-CgVPE1 fusion protein was purified using a GST-Sefinose (TM) Kit (Sangon Biotech, Shanghai, China) and dialyzed into 50 mM Tris-Cl, 0.1 M NaCl, 2 mM ethylenediaminetetraacetic acid (EDTA) at pH 8 and stored at −80 °C [58].

#### 4.2.9. Enzyme Assays

VPE activity and caspase-1-like activity were measured according to the method described by Teper-Bamnolker et al. [59]. The sample was homogenized in an extraction buffer (50 mM sodium acetate, 50 mM NaCl, 1 mM EDTA, and 100 mM dithiothreitol [DTT], pH 5.5) at 4 °C for 3 h. The solution was centrifuged at 15,000× *g* at 4 °C for 30 min, and the supernatant was used for enzyme assays. Protein content was measured using a Modified Bradford Protein Assay Kit (Sangon Biotech, Shanghai, China). Ac-ESEN-MCA (N-acetyl-Glu-Ser-Glu-Asp-4-methylcoumaryl-7-amide; Peptide Institute, Osaka, Japan) and Ac-YVAD-MCA (N-acetyl-Tyr-Val-Ala-Asp-4-methylcoumaryl-7-amide; Peptide Institute) were used as fluorogenic-specific substrates for VPE and caspase-1, respectively. Crude protein (10 μg), as mentioned above, was then incubated in a reaction buffer (100 mM sodium acetate, 100 mM DTT, 50 μM fluorogenic substrate, and 50 μM E64-d [a cysteine proteinase inhibitor, Peptide Institute], pH 5.5) at 20 °C for 4 h. The amount of 7-amino-4-methylcoumarin (AMC) released was determined spectrophotometrically at excitation and emission wavelengths of 380 and 460 nm, respectively. A known concentration of AMC were used for the standard calibration. Recombinant GST-CgVPE1 fusion protein (5 μg) was placed in a reaction buffer (containing fluorogenic substrate, pH 7.0 and 5.5) at 30 °C for 8 h. Meanwhile, 50 μM of the peptide inhibitor (Ac-YVAD-CHO) was added to the enzyme assay to ensure the specificity of the reaction [59].

#### 4.2.10. Self-Activation Assay

For self-activation, 50 μg of the crude protein, which was extracted according to the description above, or 50 ng GST-CgVPE1, was mixed into 100 μL of the buffer (0.1 M sodium acetate, 0.1 mM dithiothreitol, 5 mM cysteine, and 0.1 mM EDTA, pH 5.5 and 7.0, respectively) and incubated for 1 h at 30 °C. Western blotting was used to detect mature CgVPE1 proteins [54]. In addition, the extraction of active proteins was performed according to the description in Section 4.2.9 with a slight change. The sample was homogenized in an extraction buffer (50 mM sodium acetate, 50 mM NaCl, 1 mM EDTA, and 100 mM dithiothreitol [DTT], pH 7.0) at 4 °C for 3 h. Then, the same amount of protein was reacted in a neutral pH 7.0 buffer and acidic pH 5.5 buffer for 1 h at 30 °C, respectively. Western blotting was used to detect the mature CgVPE1 proteins.

#### 4.2.11. Statistic Analysis

The Statistical Package for the Social Sciences, SPSS 21.0 software was used for the t-tests alongside Duncan’s multiple comparisons. The Image J software (National Institutes of Health, Bethesda, MD, USA) was used to analyze the abundance of gold particles in different cell components during secretory cavity development. The figures that appear in the manuscript were typeset using Adobe Photoshop CS6 software (Adobe, San Jose, CA, USA).

## Data Availability

Not applicable.

## Supplementary Materials

The following supporting information can be downloaded at: https://www.mdpi.com/article/10.3390/ijms241411681/s1.
