# Supplementary material for: Involvement of Vacuolar Processing Enzyme CgVPE1 in Vacuole Rupture in the Programmed Cell Death during the Development of the Secretory Cavity in Citrus grandis ‘Tomentosa’ Fruits
Source: Int J Mol Sci. 2023 Jul 20;24(14):11681. doi: 10.3390/ijms241411681 (PMC10380461; doi:10.3390/ijms241411681)
Supplement: Supplementary file 1 [file ijms-24-11681-s001.zip › ijms-2468338-supplementary.pdf]

Supplementary Materials:

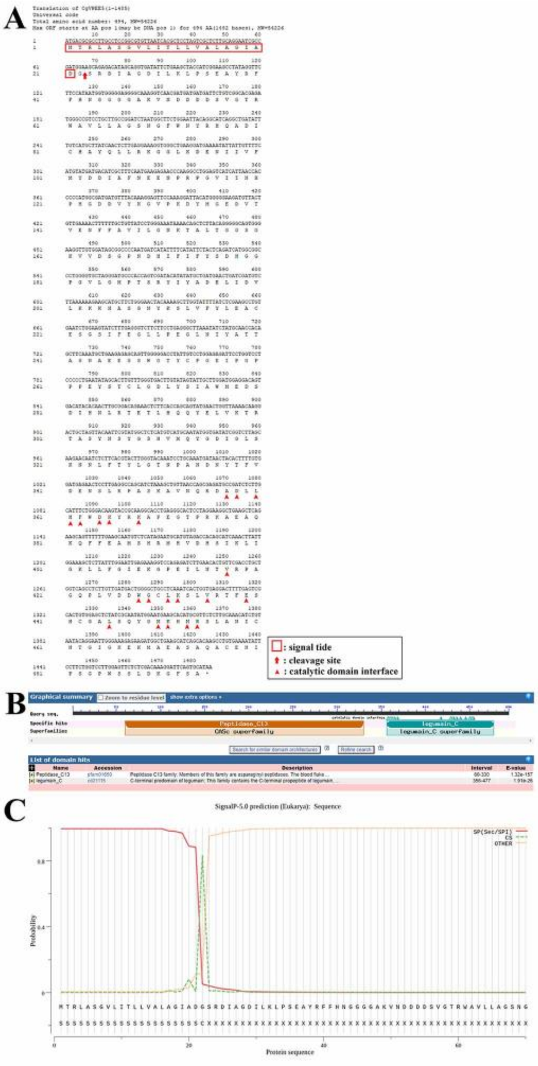

Figure S1. Sequence analysis of CgVPE1.

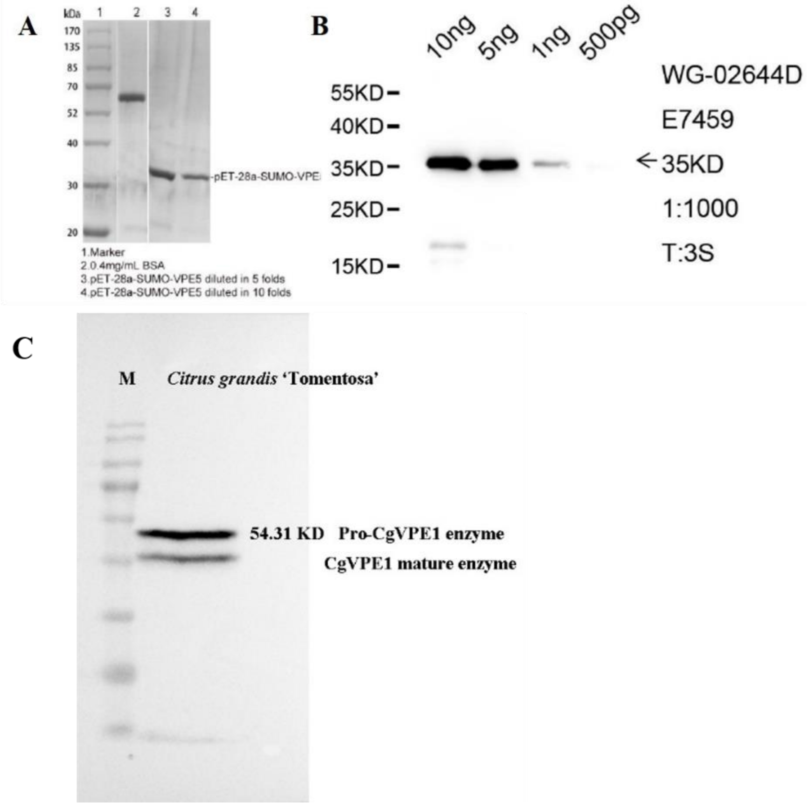

Figure S2. The Expression of CgVPE1 immunogen and exogenous verification of CgVPE1 antibody. (A) SDS-PAGE image of CgVPE1 protein 303-422aa, the antigen protein was about 35 kDa; (B) Antibody immunoassay for different amounts of protein; (C) Antibody immunoassay for the total protein of *C. grandis* 'Tomentosa'.

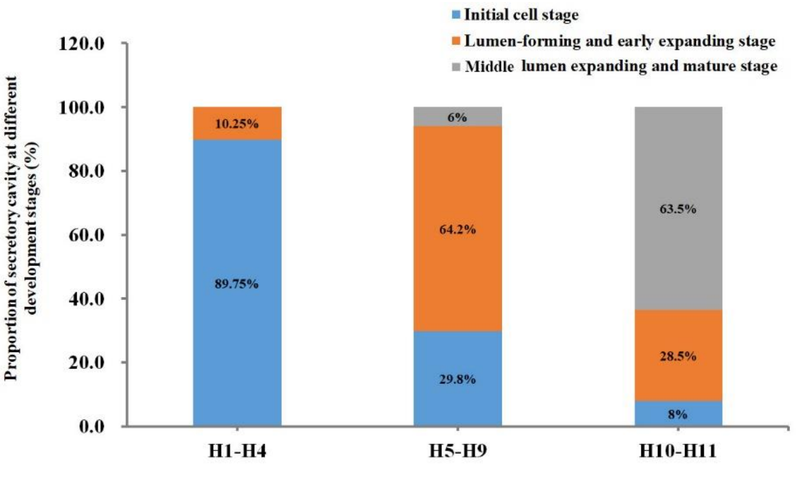

Figure S3. Statistics of the proportion of different developmental stages of secretory cavities in ovary or young fruit of different size in *C. grandis* 'Tomentosa'.

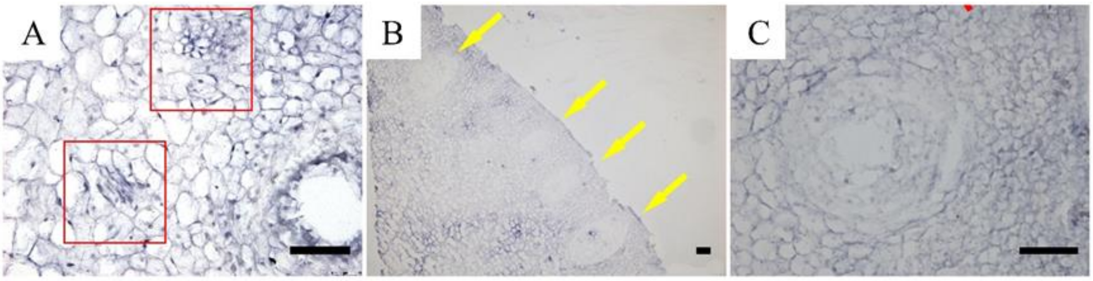

Figure S4. The negative control of In situ hybridization of *CgVPE1*
(A) *CgVPE1* is specifically expressed in the vascular bundle cells and secretory cavities; and the red box indicates the vascular bundle cells; (B-C) were the negative control. The yellow arrows point at the secretory cavity. bars=50 μm.

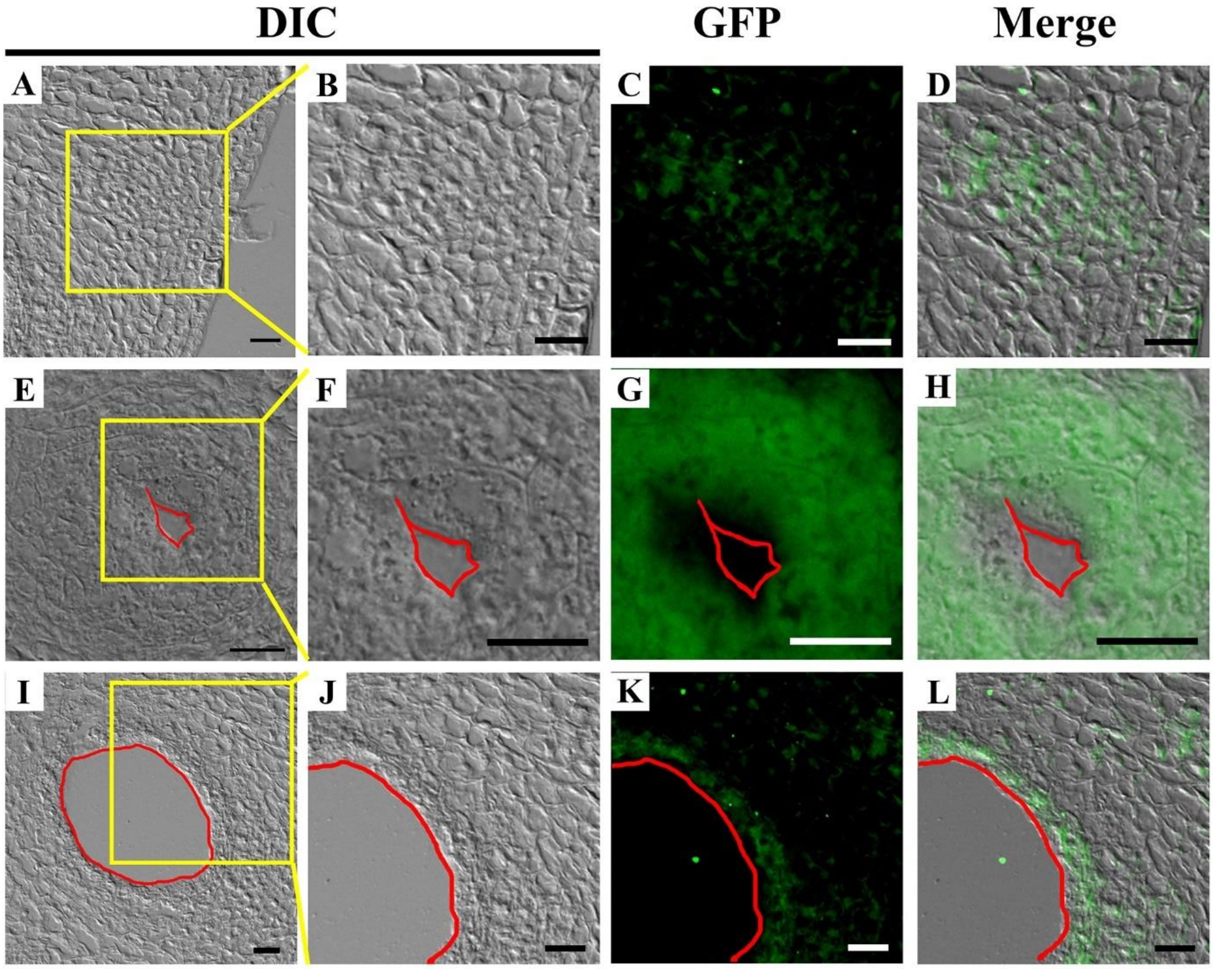

Figure S5. The immunofluorescence localization of CgVPE1. (A-D) A) the late initial cell stage. (D) is merged (B) and (C). (E-H) the lumen-forming stage, (H) is merged (G) and (F); (I-L) the middle expanding lumen stage, (L) is merged (J) and (K); the red circle indicates the lumen of secretory cavity in (E-L). Bar= 20 μm.

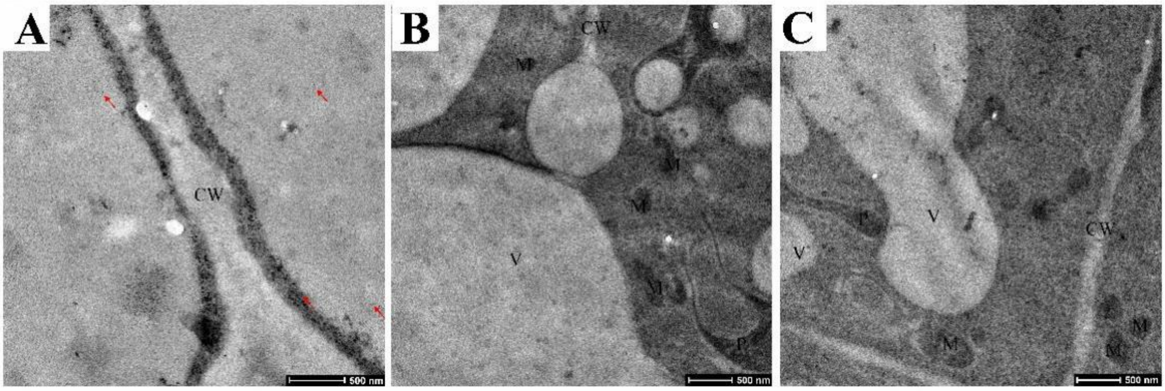

Figure S6. The control of CgVPE1 immunocytochemical localization. A, the distribution of immunogold particles of CgVPE1 in parenchyma cell vacuole; B, negative control of only incubated with secondary antibody; C, negative control of only incubated with primary antibody; red arrows points at immunogold particles in the vacuole; V, vacuole; CW, cell wall; M, mitochondria; P, plastids; A-C, bars=500 nm

A

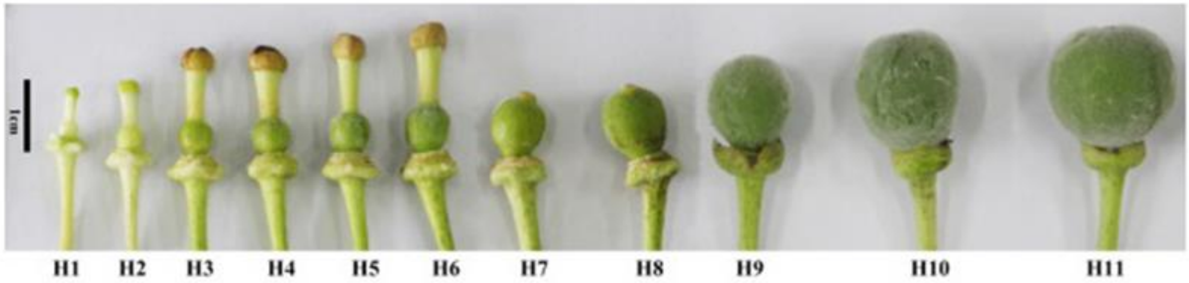

B

Length×Width (mm)	2×2	3×3	4×4	6×5	7×5	8×5	8×7	10×10	13×12	17×17	19×19
Stages	H1	H2	H3	H4	H5	H6	H7	H8	H9	H10	H11

Figure S7. The morphology and sampling standard of ovaries and young fruits of *C. grandis* 'Tomentosa'. (A) The morphology of ovaries and young fruits of *C. grandis* 'Tomentosa'; H-1, 2, 3 and 4, the unopened pistils flowers; H-5, 6 and 7, The flowers have opened; H-8, 9, 10 and11, The petals have fallen off; (B) sampling standard for ovaries and young fruits of *C. grandis* 'Tomentosa'.

Table S1. *VPE* genes obtained from the sweet orange gene database

Gene name	Locus	Description
CsiVPE1	NM_001288848.1	Citrus sinensis vacuolar-processing enzyme-like (VPE)
CsiVPE2	XM_006477190.4	*Citrus sinensis* legumain (LOC102623739)
CsiVPE3a	XM_052444192.1	*Citrus sinensis* vacuolar-processing enzyme-like (LOC127903698), transcript variant X1
CsiVPE3b	XM_052444193.1	*Citrus sinensis* vacuolar-processing enzyme-like (LOC127903698), transcript variant X2

Table S2. Prediction of subcellular localization of CgVPE1 protein

Predicted subcellular localization	Vacu	Chlo	Nucl	Mito	Plas	Extro	E.R.	Golg
Possibility	7	1	1	1	1	1	1	1

Note: The value indicates the degree of possibility; the larger the value, the greater the possibility of locating it on the structure was. Vacu, vacuole; Chlo, chloroplast; Nucl, nuclear; Mito, mitochondrial; Plas, plastids; Extro, extracellular; ER, endoplasmic reticulum; Golg, Golgi.

Table S3. Primer list

gene	primer 5'-3'
CgVPE1	F: GGATGCCCACCAGTCGATAC
	R: GGAAGAAGACCCTCAAAGATA
CgVPE2	F: TGCTGAGGAAAGGTGGGTTG
	R: TCGGCACACCGTCATAAAGA
CgVPE3a	F: TGCTTCACTTCTTCTCCTTT
	R: CCAGCAACTAGAACTACCCA
CgVPE3b	F: GGCAAGGTTGTAAATAGTGG
	R: TAGCGTAGAGGTCATCTCCAGT
c-CgVPE1	F: ATGACGCGCCTTGCCTCCGGC
	R: TTATGCACTGAATCCTTTGTCGAGA
c-CgVPE2	F: ATGCTTTTTAAATACGACTTTCTCC
	R: TCAAGCGCTATAACCTCTAA
β-actin	F: CACACTGGAGTGATGGTTGG
	R: ATTGGCCTTGGGGTTAAGAG
pGEX-CgVPE1	F：TGGATCCCCGGAATTCATGACGCGCCTTGCCTCCGGC
	R：CTCGAGTCGACCCGG TTATGCACTGAATCCTTTGTCGAGA

Table S4 Probe Sequences for in situ hybridization

Genes	Probe sequence (5'-3')
CgVPE1	ATGACGCGCCTTGCCTCCGGCGTGCTAATCACGCTCCTAGTCGCTCTTGCAGG AATCGCCGATGGAAGCAGAGACATAGCAGGTGACATTCTGAAGCTACCATCG GAAGCCTATAGGTTCTTCCATAATGGTGGGGGAGGGGCAAAGGTCAACGATG ATGATGATTCTGTCGGCACGAGATGGGCCGTCCTGCTTGCCGGATCTAATGGC TTCTGG
